# Supplementary material for: Reference Genes across Nine Brain Areas of Wild Type and Prader-Willi Syndrome Mice: Assessing Differences in Igfbp7, Pcsk1, Nhlh2 and Nlgn3 Expression
Source: Int J Mol Sci. 2022 Aug 5;23(15):8729. doi: 10.3390/ijms23158729 (PMC9369261; doi:10.3390/ijms23158729)
Supplement: Supplementary file 1 [file ijms-23-08729-s001.zip › Supplemental Figure S1-S9_fixed.pdf]

A

Expression stability in olfactory bulb between WT and PWScr$^{m+/p-}$

Method	1	2	3	4	5	6	7	8
Delta CT	Alg5	Snhg12	Tfrc	Cyc1	Hmbs	Man2b2	Mogs	Gusß
BestKeeper	Snhg12	Alg5	Tfrc	Hmbs	Mogs	Gusß	Cyc1	Man2b2
Normfinder	Alg5	Snhg12	Tfrc	Cyc1	Hmbs	Mogs	Man2b2	Gusß
Genorm	Snhg12 I Alg5		Tfrc	Hmbs	Cyc1	Man2b2	Mogs	Gusß
avg. ranking	Alg5	Snhg12	Tfrc	Hmbs	Cyc1	Mogs	Man2b2	Gusß

B

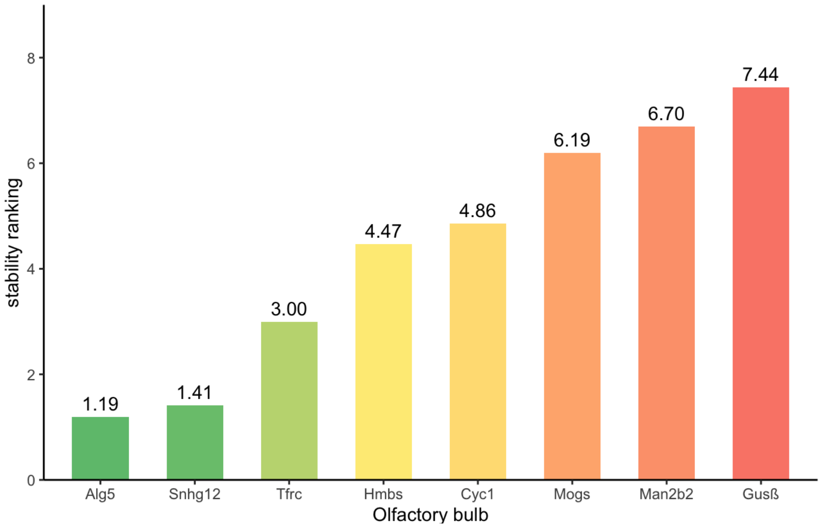

Figure S1. Reference gene ranking in olfactory bulb. (**A**) Expression stability ranking of reference gene candidates in olfactory bulb between WT and *PWScr*$^{m+/p-}$ mice; determined by four different stability ranking methods. Avg. ranking: shows the calculation of the resulting average ranking of the candidate genes (highlighted in gray; bottom row). (**B**) Average ranking position of each of the genes (lower average rank/green is more stable than higher average rank/red) in olfactory bulb between WT and *PWScr*$^{m+/p-}$ mice.

A

Expression stability in isocortex between WT and PWScr$^{m+/p-}$

Method	1	2	3	4	5	6	7	8
Delta CT	Alg5	Man2b2	Tfrc	Hmbs	Mogs	Gusß	Cyc1	Snhg12
BestKeeper	Hmbs	Alg5	Man2b2	Tfrc	Gusß	Mogs	Cyc1	Snhg12
Normfinder	Alg5	Man2b2	Tfrc	Hmbs	Mogs	Gusß	Cyc1	Snhg12
Genorm	Man2b2 I Alg5		Hmbs	Tfrc	Mogs	Cyc1	Gusß	Snhg12
avg. ranking	Alg5	Man2b2	Hmbs	Tfrc	Mogs	Gusß	Cyc1	Snhg12

B

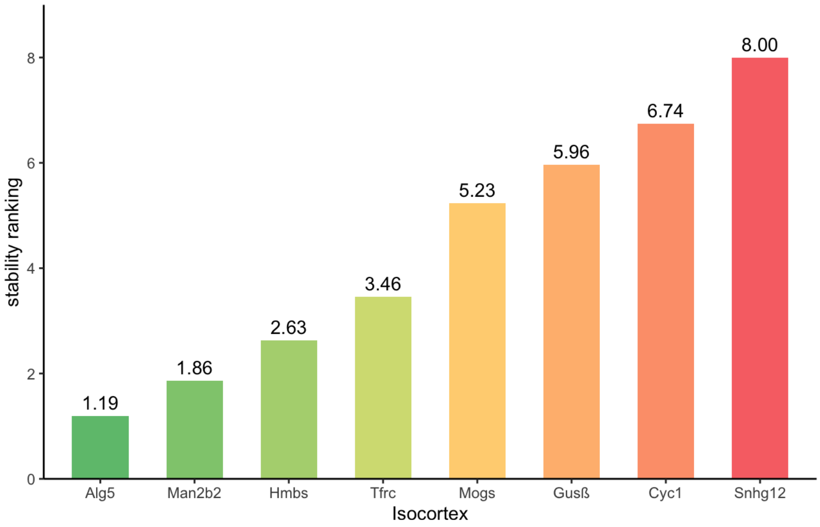

Figure S2. Reference gene ranking in isocortex. (**A**) Expression stability ranking of reference gene candidates in isocortex between WT and *PWScr*$^{m+/p-}$ mice; determined by four different stability ranking methods. Avg. ranking: shows the calculation of the resulting average ranking of the candidate genes (highlighted in gray; bottom row). (**B**) Average ranking position of each of the genes (lower average rank/green is more stable than higher average rank/red) in isocortex between WT and *PWScr*$^{m+/p-}$ mice.

A

Expression stability in hippocampus between WT and PWScr$^{m+/p-}$

Method	1	2	3	4	5	6	7	8
Delta CT	Hmbs	Alg5	Gusß	Mogs	Cyc1	Snhg12	Man2b2	Tfrc
BestKeeper	Alg5	Snhg12	Hmbs	Gusß	Mogs	Man2b2	Tfrc	Cyc1
Normfinder	Hmbs	Alg5	Gusß	Mogs	Cyc1	Snhg12	Man2b2	Tfrc
Genorm	Mogs I Hmbs		Gusß	Alg5	Cyc1	Snhg12	Man2b2	Tfrc
avg. ranking	Hmbs	Alg5	Mogs	Gusß	Snhg12	Cyc1	Man2b2	Tfrc

B

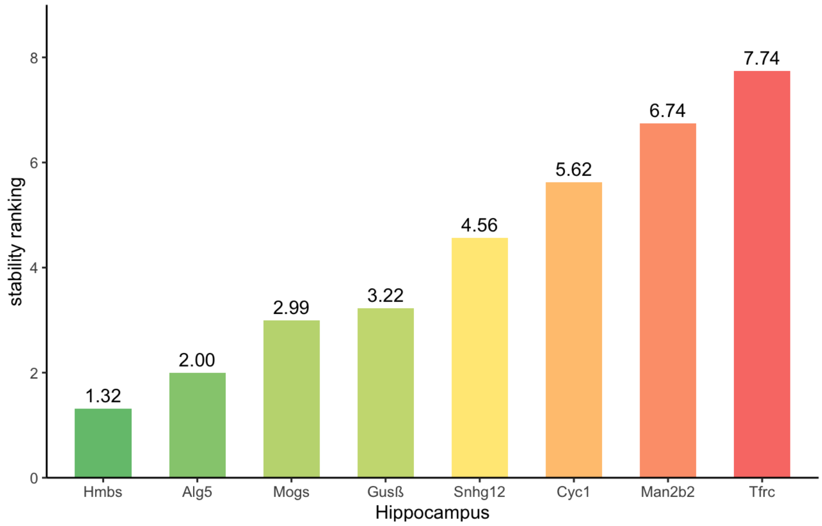

Figure S3. Reference gene ranking in hippocampus. (**A**) Expression stability ranking of reference gene candidates in hippocampus between WT and *PWScr*$^{m+/p-}$ mice; determined by four different stability ranking methods. Avg. ranking: shows the calculation of the resulting average ranking of the candidate genes (highlighted in gray; bottom row). (**B**) Average ranking position of each of the genes (lower average rank/green is more stable than higher average rank/red) in hippocampus between WT and *PWScr*$^{m+/p-}$ mice.

A

Expression stability in thalamus between WT and PWScr$^{m+/p-}$

Method	1	2	3	4	5	6	7	8
Delta CT	Tfrc	Alg5	Gusß	Cyc1	Snhg12	Hmbs	Man2b2	Mogs
BestKeeper	Alg5	Gusß	Tfrc	Hmbs	Snhg12	Mogs	Man2b2	Cyc1
Normfinder	Tfrc	Alg5	Gusß	Cyc1	Hmbs	Snhg12	Man2b2	Mogs
Genorm	Snhg12 I Cyc1		Gusß	Alg5	Tfrc	Hmbs	Man2b2	Mogs
avg. ranking	Tfrc	Alg5	Gusß	Cyc1	Snhg12	Hmbs	Man2b2	Mogs

B

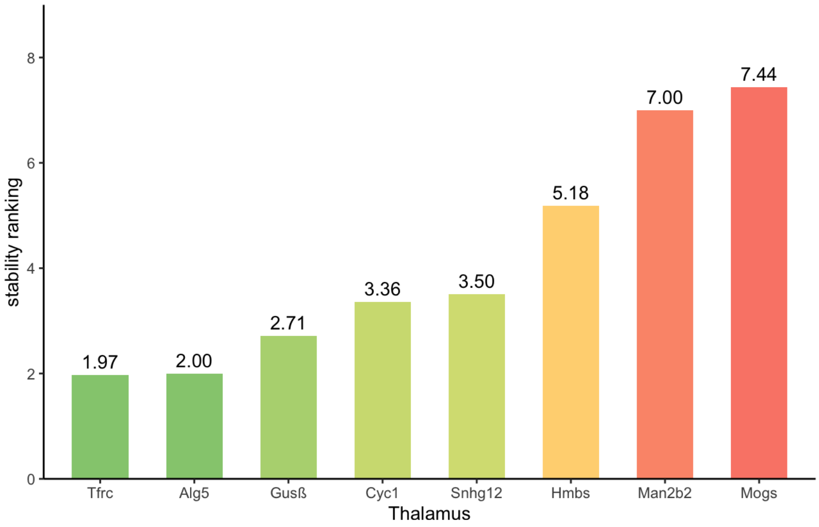

Figure S4. Reference gene ranking in thalamus. (**A**) Expression stability ranking of reference gene candidates in thalamus between WT and *PWScr*$^{m+/p-}$ mice; determined by four different stability ranking methods. Avg. ranking: shows the calculation of the resulting average ranking of the candidate genes (highlighted in gray; bottom row). (**B**) Average ranking position of each of the genes (lower average rank/green is more stable than higher average rank/red) in thalamus between WT and *PWScr*$^{m+/p-}$ mice.

A

Expression stability in hypothalamus between WT and PWScr$^{m+/p-}$

Method	1	2	3	4	5	6	7	8
Delta CT	Alg5	Hmbs	Man2b2	Gusß	Mogs	Snhg12	Tfrc	Cyc1
BestKeeper	Snhg12	Hmbs	Alg5	Mogs	Gusß	Man2b2	Tfrc	Cyc1
Normfinder	Alg5	Man2b2	Hmbs	Gusß	Mogs	Snhg12	Tfrc	Cyc1
Genorm	Gusß I Alg5		Mogs	Hmbs	Man2b2	Snhg12	Tfrc	Cyc1
avg. ranking	Alg5	Hmbs	Gusß	Man2b2	Snhg12	Mogs	Tfrc	Cyc1

B

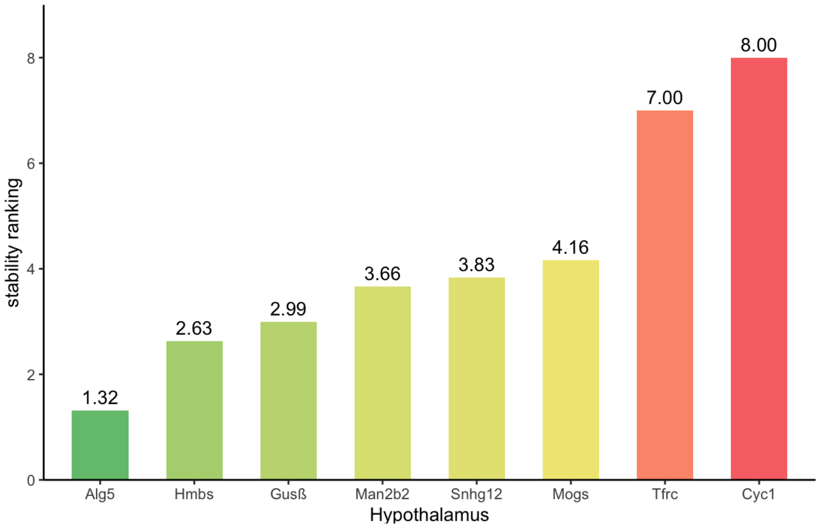

Figure S5. Reference gene ranking in hypothalamus. (**A**) Expression stability ranking of reference gene candidates in hypothalamus between WT and *PWScr*$^{m+/p-}$ mice; determined by four different stability ranking methods. Avg. ranking: shows the calculation of the resulting average ranking of the candidate genes (highlighted in gray; bottom row). (**B**) Average ranking position of each of the genes (lower average rank/green is more stable than higher average rank/red) in hypothalamus between WT and *PWScr*$^{m+/p-}$ mice.

A

Expression stability in midbrain between WT and PWScr$^{m+/p-}$

Method	1	2	3	4	5	6	7	8
Delta CT	Hmbs	Mogs	Cyc1	Snhg12	Alg5	Gusß	Tfrc	Man2b2
BestKeeper	Hmbs	Cyc1	Mogs	Gusß	Alg5	Snhg12	Man2b2	Tfrc
Normfinder	Hmbs	Cyc1	Mogs	Snhg12	Alg5	Gusß	Tfrc	Man2b2
Genorm	Mogs I Hmbs		Cyc1	Snhg12	Alg5	Gusß	Tfrc	Man2b2
avg. ranking	Hmbs	Mogs	Cyc1	Snhg12	Alg5	Gusß	Tfrc	Man2b2

B

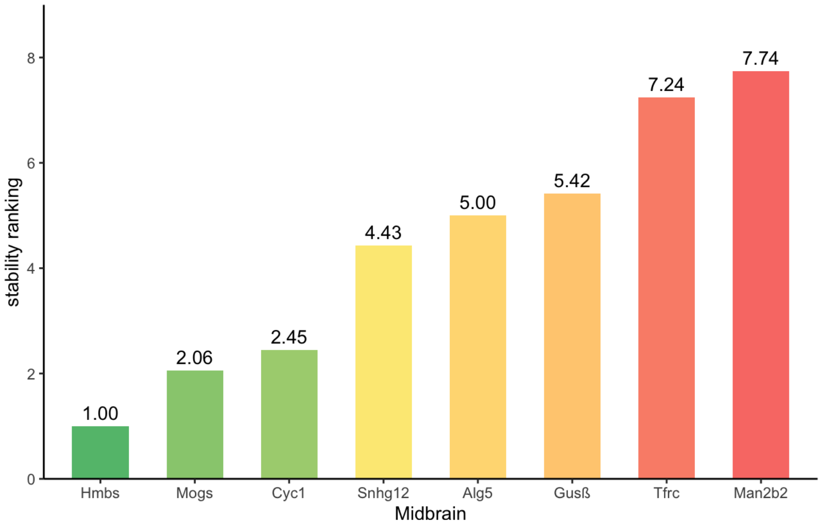

Figure S6. Reference gene ranking in midbrain. (**A**) Expression stability ranking of reference gene candidates in midbrain between WT and *PWScr*$^{m+/p-}$ mice; determined by four different stability ranking methods. Avg. ranking: shows the calculation of the resulting average ranking of the candidate genes (highlighted in gray; bottom row). (**B**) Average ranking position of each of the genes (lower average rank/green is more stable than higher average rank/red) in midbrain between WT and *PWScr*$^{m+/p-}$ mice.

A

Expression stability in cerebellum between WT and PWScr$^{m+/p-}$

Method	1	2	3	4	5	6	7	8
Delta CT	Hmbs	Alg5	Man2b2	Cyc1	Gusß	Tfrc	Mogs	Snhg12
BestKeeper	Tfrc	Snhg12	Hmbs	Man2b2	Alg5	Mogs	Gusß	Cyc1
Normfinder	Hmbs	Alg5	Man2b2	Cyc1	Gusß	Tfrc	Mogs	Snhg12
Genorm	Hmbs I Alg5		Cyc1	Man2b2	Gusß	Tfrc	Mogs	Snhg12
avg. ranking	Hmbs	Alg5	Man2b2	Tfrc	Cyc1	Gusß	Snhg12	Mogs

B

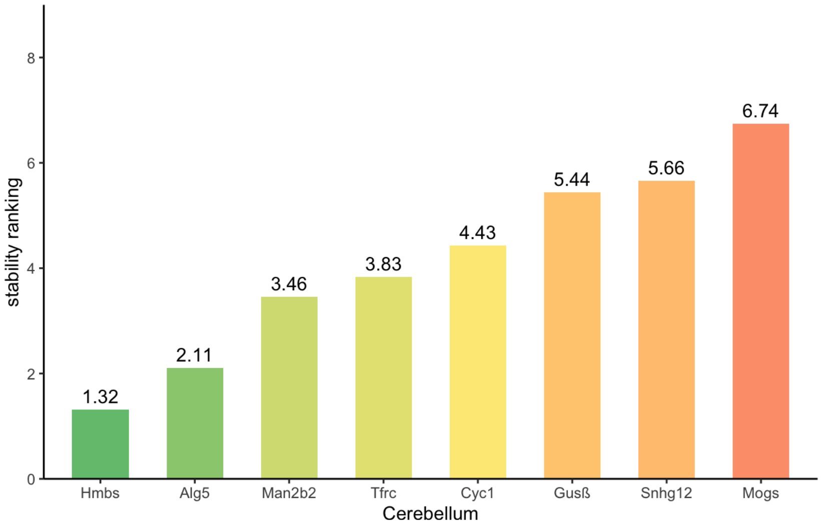

Figure S7. Reference gene ranking in cerebellum. (**A**) Expression stability ranking of reference gene candidates in cerebellum between WT and *PWScr*$^{m+/p-}$ mice; determined by four different stability ranking methods. Avg. ranking: shows the calculation of the resulting average ranking of the candidate genes (highlighted in gray; bottom row). (**B**) Average ranking position of each of the genes (lower average rank/green is more stable than higher average rank/red) in cerebellum between WT and *PWScr*$^{m+/p-}$ mice.

A

Expression stability in pons between WT and PWScr$^{m+/p-}$

Method	1	2	3	4	5	6	7	8
Delta CT	Mogs	Cyc1	Hmbs	Snhg12	Alg5	Gusß	Man2b2	Tfrc
BestKeeper	Alg5	Man2b2	Cyc1	Snhg12	Tfrc	Hmbs	Gusß	Mogs
Normfinder	Mogs	Hmbs	Cyc1	Alg5	Snhg12	Gusß	Man2b2	Tfrc
Genorm	Snhg12 I Gusß		Mogs	Cyc1	Alg5	Hmbs	Man2b2	Tfrc
avg. ranking	Mogs	Cyc1	Snhg12	Alg5	Hmbs	Gusß	Man2b2	Tfrc

B

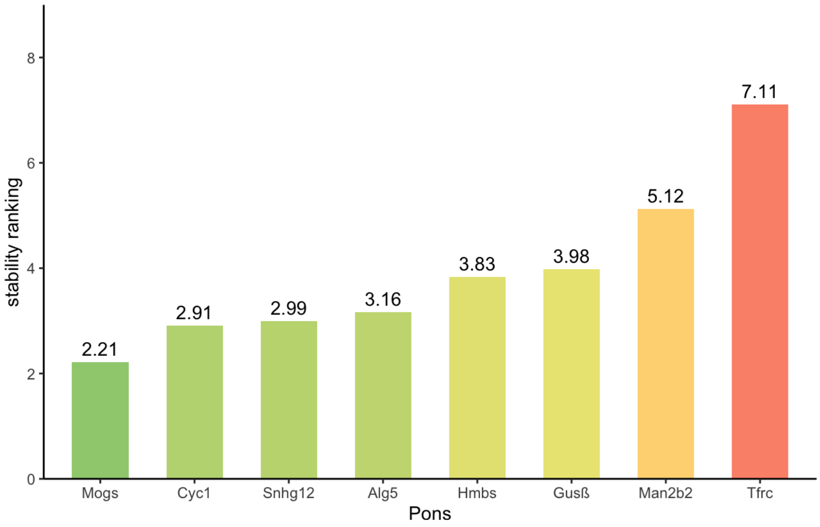

Figure S8. Reference gene ranking in pons. (**A**) Expression stability ranking of reference gene candidates in pons between WT and *PWScr*$^{m+/p-}$ mice; determined by four different stability ranking methods. Avg. ranking: shows the calculation of the resulting average ranking of the candidate genes (highlighted in gray; bottom row). (**B**) Average ranking position of each of the genes (lower average rank/green is more stable than higher average rank/red) in pons between WT and *PWScr*$^{m+/p-}$ mice.

A

Expression stability in medulla between WT and $PWScr^{m+/p-}$

Method	1	2	3	4	5	6	7	8
Delta CT	Hmbs	Alg5	Man2b2	Gusß	Cyc1	Snhg12	Tfrc	Mogs
BestKeeper	Hmbs	Alg5	Man2b2	Mogs	Snhg12	Tfrc	Gusß	Cyc1
Normfinder	Hmbs	Alg5	Man2b2	Gusß	Cyc1	Tfrc	Snhg12	Mogs
Genorm	Snhg12 I Alg5		Gusß	Hmbs	Cyc1	Tfrc	Man2b2	Mogs
avg. ranking	Hmbs	Alg5	Man2b2	Snhg12	Gusß	Cyc1	Tfrc	Mogs

B

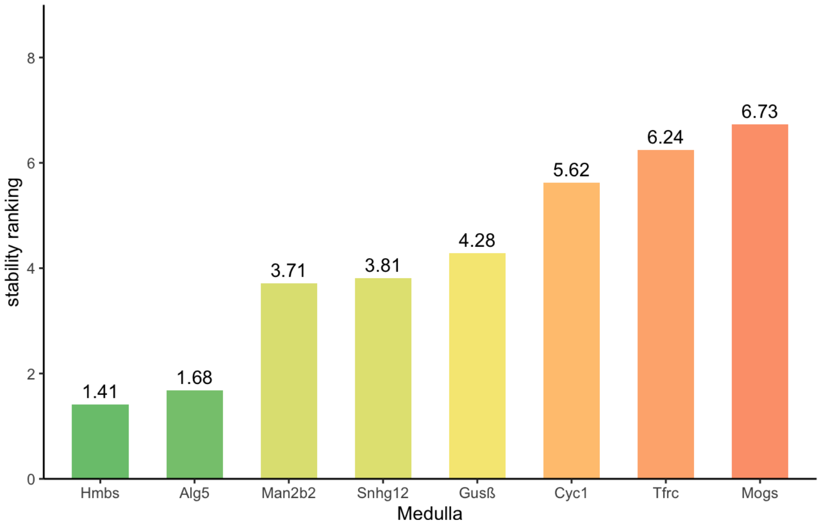

Figure S9. Reference gene ranking in medulla. (**A**) Expression stability ranking of reference gene candidates in medulla between WT and *PWScr*$^{m+/p-}$ mice; determined by four different stability ranking methods. Avg. ranking: shows the calculation of the resulting average ranking of the candidate genes (highlighted in gray; bottom row). (**B**) Average ranking position of each of the genes (lower average rank/green is more stable than higher average rank/red) in medulla between WT and *PWScr*$^{m+/p-}$ mice.
