# Supplementary material for: Reference Genes across Nine Brain Areas of Wild Type and Prader-Willi Syndrome Mice: Assessing Differences in Igfbp7, Pcsk1, Nhlh2 and Nlgn3 Expression
Source: Int J Mol Sci. 2022 Aug 5;23(15):8729. doi: 10.3390/ijms23158729 (PMC9369261; doi:10.3390/ijms23158729)
Supplement: Supplementary file 1 [file ijms-23-08729-s001.zip › Kummerfeld_et_al_TableS1.pdf]

RT-qPCR analysis						
Gene symbol - name			Primer sequences	Amplicon	Probe #	Probe sequence
ActB	Beta-actin	F	ctaaggccaaccgtgaaaag	104 nt	#64	ccaggctg
		R	accagaggcatacagggaca			
Alg5	Dolichyl-phosphate beta-glucosyltransferase	F	cacagtgcgggttcaaattac	96 nt	#66	ggctgctg
		R	acagcagctctacatcaaatgc			
B2M	Beta-2-Microglobulin		Thermofisher, Assay ID Mm00437762_m1			
Cyc1	Cytochrome c1	F	tgctacacggaggaagaagc	71 nt	#10	ccacctcc
		R	ccatcatcattagggccatc			
Gusß	Beta-glucuronidase	F	gaggatcaacagtgcccatt	91nt	#31	ttccacca
		R	agcctcaaaggggaggtg			
Hmbs	Mydroxymethylbilane synthase	F	agaaaagtgccgtgggaac	99 nt	#91	ctctcctc
		R	tgttgaggtttccccgaat			
Man2b2	Mannosidase Alpha Class 2B Member 2	F	tgactacgacctcaaggatgc	62 nt	#4	cttcctgc
		R	acccatgccacacgaact			
Mogs	Mannosyl-Oligosaccharide Glucosidase	F	gctcaccactgagttcgtca	108 nt	#78	ctccagct
		R	accaaagggaaggaaggtgt			
Sdha	Succinate Dehydrogenase Complex Flavoprotein Subunit A	F	ccctgagcattgcagaatc	70 nt	#80	ggcggcgg
		R	tcttctccagcatttgcctta			
Snhg12	Small Nucleolar RNA Host Gene 12	F	ggaagggaccggatttttc	112 nt	#93	ggaccaga
		R	cctcacaatcttcatcaacagg			
Tfrc	Transferrin receptor	F	gctttgggtgctggtgtt	109 nt	#47	tccagtgt
		R	tgctgggtctaaatccatcttt			
Igfbp7	insulin-like growth factor binding protein 7	F	tgccctccatgaaataccac	96 nt	#110	AGCCTCTG
		R	ggctgtctgagagcaccttt			
Pcsk1	proprotein convertase subtilisin/kexin type 1	F	tggagttgcatataattccaaagtt	73 nt	#42	GCTGGATG
		R	agcctcaatggcatcagttac			
Pcsk2	proprotein convertase subtilisin/kexin type 2	F	aggaagaggctcgccaag	78 nt	#84	TCTGCTGC
		R	agtgatacaggccttctgcaa			
Nhlh2	nescient helix loop helix 2	F	aatattggctgcttttaatatttgc	125 nt	#63	CTCCTCCT
		R	ggactcagcatcattttggag			
Nlgn3_total	Neuroligin 3	F	CCACGGAGGCTCTTACATGG	135 nt	#121	GATGGCAG
		R	GCCTGATCTCCAGTGCTCAG			
Nlgn3_Iso1	Neuroligin 3 (isoform 1, incl. Exon3)	F	GCCCAACAAGAAAATTTGTAG(	203 nt	#147	GCCATCAA
		R	TGACGTTGCCGTAACTTGC			
Nlgn3_Iso2	Neuroligin 3 (isoform 2, excl. Exon3)	F	TGCCCACGGAAGATGGATC	193 nt	#147	GCCATCAA
		R	ACGATGACGTTGCCGTAACT			

DNA oligonucleotides for northern blot hybridization

Snord115R - CAGCGTAATCCTATTGAGCATGAAT
Snord116R - GTTCCGATGAGAGTGGCGGTACAGAGT

Table S1. List of oligonucleotides primers and probes used in RT-PCR analyses and Northern blot hybridization.
